# Effects of soaking seeds in exogenous vitamins on active oxygen metabolism and seedling growth under low-temperature stress

**DOI:** 10.1016/j.sjbs.2021.02.065

**Published:** 2021-03-02

**Authors:** Yu Xin Chi, Li Yang, Chang Jiang Zhao, Ihsan Muhammad, Xun Bo Zhou, Hong De Zhu

**Affiliations:** a College of Agronomy, Heilongjiang Bayi Agricultural University, Daqing 163319, China; b Agricultural College of Guangxi University, Nanning 530004, China

**Keywords:** Active oxygen metabolism, Exogenous vitamins, Low-temperature stress, Maize

## Abstract

This study investigated the influence of the exogenous application of vitamin B2 (V_B2_), B12 (V_B12_), biotin (V_H_), and nicotinic acid (V_PP_) on oxygen production in maize (*Zea mays* L.) seedlings at 5 °C for day 1, 3, 5 and 7. The seeds were soaked in V_B2_, V_B12_, V_H_, and V_PP_ solutions for 24 h at the concentration of 100 mg/L, and control was soaked in distilled water. A total of 50 seeds were used for each treatment in germination boxes was repeated three times. The germination box was placed in a hypothermic incubator for 1, 3, 5, and 7 days in the dark at 5 °C, then moved to a plant growth room and kept for seven days. Compared with the V_H_ and V_PP_ treatments, the V_B2_ and V_B12_ treatments had higher thiobarbituric acid reactive substances, proline, and soluble sugars. The V_B2_ and V_B12_ treatments also increased the activities of superoxide dismutase (SOD), peroxidase (POD), catalase (CAT), and ascorbate peroxidase (APX) than other treatments. The V_B2_ and V_B12_ treatments reduced the contents of hydrogen peroxide (H_2_O_2_^–^), superoxide anion (O_2_^–^), and the damage of reactive oxygen species (ROS) to cells, increased the stability of the cell membrane and the content of cell osmoregulation substances. Moreover, V_B2_ and V_B12_ had higher seedling growth, germination rate, and index. Treatments V_B2_ and V_B12_ could promote maize seed germination and growth under low-temperature stress. Exogenous vitamins in crop production can be a valuable tool for protecting plants against low-temperature stress.

## 1 Introduction

Maize (*Zea mays* L.) is a thermophilic crop; however, low-temperature is expected during early spring in Northeast China (Yang et al., 2021). Low temperature is a crucial limiting factor of crop production potential in Northern China, which may damage seed germination and seedling growth. Temperature below 15 °C can induce chilling stress at the maize seedling stage (Holá et al., 2003, Pál and Nagy, 2002). A researcher demonstrated that plants growth and development are inhibited by low temperatures (Chinnusamy et al., 2007). When plants are exposed to low temperatures, water metabolism, photosynthesis, nutrient metabolism, biofilm production, and other physiological processes are negatively affected (Savitch et al., 2009). Low temperatures can reduce water transportation through stomatal restriction and disrupt cells metabolic balance and hence damage cell membranes (Aroca et al., 2003, Korkmaz et al., 2010). Moreover, low-temperatures are also reducing cellular respiration and producing ROS in plants (Sugie et al., 2006, Suzuki and Mittler, 2006).

Continuous oxidative stress triggers the enzymatic and nonenzymatic antioxidant defense systems in plants (Ashraf, 2009). Due to low-temperature stress, the ROS scavenged enzymes such as SOD, POD, CAT, and APX (Balestrasse et al., 2010, Duan et al., 2012). However, low-temperature adversely affects soil microbial biomass and community, which decreased soil mineralization and fertility (Muhammad et al., 2018, Muhammad et al., 2019). Plants have biological mechanisms to reduce the ROS-induced damage, while in low-temperatures, the ROS can damage membrane lipids, proteins, and nucleic acid, which lead to cell death (Apel and Hirt, 2004). The intracellular substances easily penetrate the surrounding environment when the membrane is damaged, increasing cell conductivity (Tang et al., 2005). Increased synthesis of osmolytes, such as soluble proteins and proline, reduces cell stress (Sarropoulou et al., 2012). Severe stress for extended periods can cause serious damage and cell death (Turk et al., 2014). The mechanism of reducing ROS damage is crucial to maintained redox balance in cells (Ahmad et al., 2020a, Ahmad et al., 2019). The accumulation of ions and osmolytes reduces the osmotic and water potential of cells, which leads to absorb water and maintain normal plant growth under adverse conditions (Singh and Usha, 2003). It has been reported that biological stimulants have positive effects on plants physiological processes (Ahmad et al., 2020b, Kamran et al., 2018). The antioxidant system can protect plants from oxidative damage caused by cold stress (Imahori et al., 2008).

Vitamins are necessary to the human body and are also synthesized by plants. Some vitamins act as signals to regulate the carbohydrate content and enzyme activity of cell metabolisms, which control plants gene expression. A previous study reported that soaking sweet corn seeds in vitamin solution had a positive effect on SOD, POD, and growth of maize seedlings and enhanced the antioxidant capacity and reduced membrane peroxidation in seedlings compared to control (Li et al., 2014). Exogenous vitamins play a vital role in producing and eliminating ROS and the expression of ROS-responsive genes (Nakano and Asada, 1981, Savitch et al., 2009). Vitamin C (V_C_) is an essential free radical scavenger in plant cells that can directly participate in the scavenging system of O_2_^–^ and maintain the reducing state of another antioxidant, vitamin E (V_E_).

Most of these studies used artificial simulation test methods, but these results are difficult to apply to cold regions with complex conditions. Information on precise vitamin applications to maize crops is limited. In our experiment, we chose an optimum concentration (100 mg/L) of exogenous vitamins for seed soaking and kept for 1, 3, 5, and 7 days at 5 °C. This study aimed to assess the effect of different kinds of exogenous vitamins on the active oxygen metabolism of maize plants and to determine the types of vitamins that could be used to promote the growth of maize crops under low-temperature stress. This research mainly focused on the oxidative system, including ROS accumulation, antioxidant enzyme activities, and soluble sugar and proline contents.

## 2 Materials and methods

### 2.1 Experimental materials and design

This experiment was conducted in 2017. Uniform, plump-eared and undamaged maize (Zhengdan 958) seeds were soaked for 10 min in a 10% sodium hypochlorite solution and then washed three times with distilled water and air-dried (Li Zhenlun et al., 2014). The seeds were soaked with V_B2_, V_B12_, V_H_, and V_PP_ solutions (100 mg/L) for 24 h and soaked with distilled water for the control treatment (CK) following the procedure of Li Zhenlun et al. (2014). A total of 50 seeds were used in the germination box and repeated three times. The seeds were uniformly sown in the germination box, placed in a hypothermic incubator for 1, 3, 5, and 7 days in the dark at 5 °C, then moved to a plant growth chamber for seven days. After10 days maize seedling were collected, and their germ and radicle lengths, weights, root-shoot ratio, germination rate and index were measured.

### 2.2 Seedling observations and measurements

#### 2.2.1 Seedling growth

The length of the germ and radicle of 10 seeds in each treatment were measured. Besides, the maize's fresh germ and radicle were separated and weighed with a sensitive electronic balance. The germ and radicle samples were then placed in an oven at 80 °C for 15 min and dried to constant weight; the root-shoot ratio is the ratio between underground dry weight and aboveground dry weight.

#### 2.2.2 Germination rate and index

The germinated seedlings in each treatment were recorded daily till 99% emergence of seeds. The germination rate and germination index were calculated.

$$Germination\;rate\left(\%\right)=\frac{Germinated\;seedlings}{Number\;of\;total\;seeds}\times 100$$

$$Germination\;index\left(\%\right)=\frac{Germinated\;seedling\;during\;first\;3\;days}{Number\;of\;total\;seeds}\times 100$$

### 2.3 Determination of oxidation parameters

Frozen plant tissues (0.5 g) were milled and homogenized with 10 mL of 0.1% (w/v) trichloroacetic acid (TCA). The homogenate was shaken for 30 min (150 rpm) and clarified by centrifugation at 5000 *g* for 15 min. The obtained supernatant was used to determine the oxidation parameters. Oxidation parameters were measured in triplicate.

#### 2.3.1 Hydrogen peroxide

The homogenate was centrifuged at 12000 *g* for 15 min, and 0.5 mL of the supernatant was added to 0.5 mL of 10 mM potassium phosphate buffer (pH 7.0) and 1 mL of 1 M potassium iodide. The absorbance was measured at 390 nm by using a UV-1800 spectrophotometer (Shimadzu Instrument, Co., Ltd, Suzhou), and the hydrogen peroxide (H_2_O_2_) content was determined according to the Velikova et al. (2000).

#### 2.3.2 Thiobarbituric acid reactive substances (TBARS)

The TBARS content was determined, as described by Hodges et al. (1999). Two milliliters of extract were briefly mixed with an equal volume of a 20% TCA solution containing 0.5% (w/v) thiobarbituric acid. The mixtures were incubated in a hot water bath (95 °C) for 30 min and centrifuged at 10000 *g* and 4 °C for 10 min. The absorbance was measured at 450 nm, 532 nm, and 600 nm.

#### 2.3.3 Superoxide anions

The superoxide anion (O_2_^–^) content was determined using the method described by Elstner and Heupel (Elstner, 1976). Frozen leaf tissues (0.5 g) were homogenized in 3 mL of potassium phosphate buffer (pH 7.8) in an ice bath, filtered, and centrifuged at 8000 *g* and 4 °C for 10 min. Two milliliters of supernatant were added to 0.5 mL of potassium phosphate buffer (50 M, pH 7.8) and 0.1 mL of a hydroxylamine hydrochloride solution (10 M), shaken, and kept at 25 °C for 20 min. One milliliter amino benzene sulfonic acid solution (58 M) and 1 mL of an α-naphthylamine solution (7 M) were added, shaken, and kept at 30 °C for 30 min. Then 1 mL of chloroform was added to extract pigments. The mixture was centrifuged at 10000 *g* and 4 °C for 10 min. The upper pink supernatant was collected, and the absorbance was measured at 530 nm.

### 2.4 Soluble osmolytes

#### 2.4.1 Soluble sugar

Soluble sugar was extracted and analyzed according to the method described by Ci et al. (2009). Frozen leaf tissues (0.5 g) were homogenized in 10 mL of distilled water in an ice bath, reacted in a boiling water bath for 20 min, and then 100 mL of the supernatant was placed into a flask. One milliliter of the extract was mixed with 5 mL of an anthrone solution (1 g of anthrone was dissolved in 1000 mL of 80% concentrated sulfuric acid). The mixture was incubated in a hot water bath (95 °C) for 10 min. The absorbance was measured at 620 nm.

#### 2.4.2 Proline

The proline content was determined by the method of Bates et al. (1973). Leaf tissues (0.2 g) were added to 4 mL of sulfosalicylic acid (3%) and centrifuged at 10,000*g* for 30 min. Two milliliters of supernatant were placed in a test tube, and 2 mL of glacial acetic acid and 2 mL of ninhydrin reagent were added. The mixture was boiled in a water bath at 100 °C for 30 min. After cooling, 4 mL of toluene was added, and the mixture was vortexed for 30 s. The upper phase containing proline was measured using spectrophotometrically at 520 nm with toluene as a blank.

### 2.5 Total soluble protein and antioxidant enzyme activities

One gram of leaf tissue was homogenized with 10 mL of potassium phosphate buffer (0.1 M, pH 7.0), containing 0.1 mM EDTA-Na_2_, 0.5 mM ascorbate, and 1% polyvinylpolypyrrolidone (PVPP) in an ice bath. The homogenate was centrifuged at 28710*g* at 4 °C for 10 min. The supernatant was used for the determination of the antioxidant enzyme activity and protein content.

#### 2.5.1 Superoxide dismutase (SOD)

The SOD activity was measured according to Giannopolitis and Ries (Giannopolitis and Ries, 1977). Twenty microliters of enzyme solution were mixed with 3 mL of SOD reaction solution (pH 7.8, 1.5 mL of phosphate buffer, 0.3 mL of 750 M NBT, 0.3 mL of 130 mM MET, 0.3 mL of 20 M FD, 0.3 mL of 100 M EDTA-Na_2_, and 0.3 mL distilled water). The enzyme solution and control were placed in an incubator at 4000 lux for 30 min. The blank was placed in the dark, and the samples were measured at 560 nm.

#### 2.5.2 Peroxidase (POD)

The POD activity was determined, according to Hernandez et al. (2000). Twenty microliters of enzyme solution were mixed with 3 mL of a POD reaction solution (1.4 μL of guaiacol, 0.85 μL of 30% H_2_O_2_, and 0.1 M pH 6.0 phosphate buffer). The absorbance values were recorded once every 30 s at 470 nm.

#### 2.5.3 Catalase (CAT)

The CAT activity was measured using the method described by Aebi (1984). The reaction mixture contained 50 mM phosphate buffer (pH 7.0) and 15 mM H_2_O_2_. The absorption of the mixture was measured at 260 nm.

#### 2.5.4 Ascorbate peroxidase (APX)

The APX activity was determined, according to Nakano and Asada (1981). The reaction mixture consisted of 0.5 mM ASA, 0.1 mM H_2_O_2_, 0.1 mM EDTA, 50 mM sodium phosphate buffer (pH 7.0), and 0.15 mL of an enzyme solution.

#### 2.5.5 Soluble protein content

The soluble protein content was determined by following the method of Bradford (1976), using bovine serum albumin as a standard, and expressed as mg g^−1^ FW. Leaf tissues (0.2 g) were added to 2 mL of distilled water and ground into a homogenate, then washed and grind with 6 mL of distilled water and collected in the same centrifuge tube centrifuge at 10000*g* for 10 min. Collect the supernatant and dilute to 10 mL, take 100 µL of the supernatant, add 5 mL of Coomassie Brilliant Blue, and measure the colorimetric value at 595 nm.

### 2.6 Ascorbic acid content

The ascorbic acid (ASA) content was determined using the method described by Kampfenkel et al. (1995). Leaf tissues (0.5 g) were homogenized in 2.5 mL of sulfosalicylic acid (5%) and centrifuged at 10000 *g* and 4 °C for 10 min. 100 µL supernatant, add 24 µL 1.84 mol L^-1^ triethanolamine to neutralize the test solution, add 250 µL 50 mmol L^−1^ potassium phosphate buffer (pH 7.5, containing 2.5 mmol L^−1^ EDTA), incubate at 25 °C for 10 min, add 100 µL distilled water and mix well, add TCA (10%), phosphoric acid (44%), bipyridine (4%) each 200 µL and mix well, then add FeCl_3_ (3%) 100 µL and mix well, then 40 ℃ water bath for 1 h and measure the colorimetric value at 525 nm.

### 2.7 Statistical analysis

The data were checked for normality test following the Shapiro-Wilk test, and the data were found normally distributed. One-way analysis of variance was conducted with SPSS 21.0 software (SPSS Inc., Chicago, IL, USA). The least significant differences test was used to separate means and interactions. Statistical significance was evaluated at *P* ≤ 0.05.

## 3 Results

### 3.1 Seedling growth

The germ length, radicle length, germ weight, and radicle weight of maize plants were higher in V_B2_ and V_B12_ treatment compared to CK. The results showed that V_B12_ significantly increased the germ length and fresh weight of maize seedling compared with CK (*p* > 0.05). The results showed that the germ length and fresh weight of seedlings in the V_B12_ treatment were 50.45 and 46.32% higher than those in the CK treatment. The germ length, germ weight, radicle length, and weight were lower on days 1 and 3 in the V_H_ and V_PP_ treatments than CK. Likewise, at day 7 the germ length, radicle length, germ fresh weight, and radicle weight of maize plants in the V_PP_ treatment was 14.24, 17.84, 57.14, and 33.33% lower compared to CK, respectively. No significant difference was observed between V_H_ and V_PP_ at the same time under low-temperature stress. The root-shoot ratio of V_PP_ was significantly lower than CK, V_B2,_ and V_B12_ on days 3 and 5 (*P* < 0.05). Whereas the root-shoot ratio for V_B2_ was markedly higher at days 1, 3, 5, and 7 by 61.53, 34.15, 45.71, 19.05% than CK, respectively (Table 1). Likewise, the root-shoot ratio for V_B12_ was significantly higher at days 5, and 7 by 62.86, and 54.76% than CK, respectively.

**Table 1:** Germ and radicle lengths, germ and radicle weights, and root-shoot ratio of single maize seedlings under low-temperature stress and vitamin treatment.

**Treatment**	**Germ length**	**Radicle length**	**Germ fresh weight**	**Radicle fresh weight**	**Root-shoot ratio**
	**(cm)**	**(cm)**	**(mg)**	**(mg)**	
1 d					
CK	3.18 ± 0.57ab	7.73 ± 0.38b	0.14 ± 0.01b	0.06 ± 0.00c	0.39 ± 0.03b
V_B2_	4.19 ± 0.53ab	13.29 ± 0.93a	0.28 ± 0.04a	0.17 ± 0.01a	0.63 ± 0.07a
V_B12_	4.81 ± 0.39a	13.33 ± 0.56a	0.23 ± 0.00a	0.11 ± 0.00b	0.49 ± 0.04ab
V_H_	2.64 ± 0.39b	6.79 ± 0.24b	0.13 ± 0.01b	0.06 ± 0.00c	0.50 ± 0.06ab
V_PP_	3.09 ± 0.63ab	7.40 ± 0.60b	0.13 ± 0.02b	0.06 ± 0.00c	0.44 ± 0.03b
3 d					
CK	3.33 ± 0.27b	6.39 ± 0.14b	0.11 ± 0.00c	0.05 ± 0.00b	0.41 ± 0.05bc
V_B2_	4.07 ± 0.15ab	8.99 ± 0.19a	0.17 ± 0.01b	0.09 ± 0.00a	0.55 ± 0.03a
V_B12_	5.01 ± 0.23a	9.35 ± 0.38a	0.23 ± 0.03a	0.13 ± 0.02a	0.54 ± 0.05ab
V_H_	3.19 ± 0.41ab	6.14 ± 0.10b	0.10 ± 0.00c	0.03 ± 0.00b	0.33 ± 0.04c
V_PP_	3.30 ± 0.36b	6.11 ± 0.29b	0.09 ± 0.00c	0.03 ± 0.00b	0.33 ± 0.03c
5 d					
CK	3.36 ± 0.22b	6.45 ± 0.06c	0.12 ± 0.00b	0.04 ± 0.00b	0.35 ± 0.01bc
V_B2_	4.27 ± 0.20a	9.44 ± 0.46a	0.21 ± 0.00a	0.11 ± 0.00a	0.51 ± 0.02a
V_B12_	3.39 ± 0.24b	8.87 ± 0.99ab	0.20 ± 0.01a	0.12 ± 0.01a	0.57 ± 0.01a
V_H_	3.66 ± 0.24b	7.30 ± 0.60bc	0.11 ± 0.00b	0.04 ± 0.00b	0.40 ± 0.05b
V_PP_	3.33 ± 0.10b	7.60 ± 0.13abc	0.12 ± 0.00b	0.03 ± 0.00b	0.29 ± 0.04c
7 d					
CK	3.53 ± 0.41b	6.67 ± 0.68bc	0.11 ± 0.00c	0.04 ± 0.00b	0.40 ± 0.02b
V_B2_	5.47 ± 0.78a	9.63 ± 0.39a	0.23 ± 0.01a	0.11 ± 0.00a	0.46 ± 0.04b
V_B12_	4.52 ± 0.43ab	8.49 ± 0.52ab	0.18 ± 0.02b	0.10 ± 0.00a	0.59 ± 0.06a
V_H_	3.93 ± 0.38ab	7.37 ± 0.49bc	0.10 ± 0.00c	0.04 ± 0.00bc	0.37 ± 0.02b
V_PP_	3.09 ± 0.25c	5.66 ± 0.76c	0.07 ± 0.00c	0.03 ± 0.00c	0.39 ± 0.02b

Control (CK), vitamin B2 (V_B2_), vitamin B12 (V_B12_), biotin (V_H_), and nicotinic acid (V_PP_). Values with different letters in the same column are significantly different at *P* < 0.05 based on the *Duncan* test; data are represented as the mean of three replicates ± *SE.*

### 3.2 Germination rate and index

The germination rate of maize plants in the V_B2_, V_B12_, V_H_, and V_PP_ treatments was higher than CK. The germination rate for V_B2_ and V_B12_ was higher than other treatments of the group, which resulted in 47.92 and 59.06% higher germination at day 1 and 48.48 and 68.7% at day 3 as compared to CK, respectively. The germination rate of V_H_ and V_PP_ treatments decreasing with increasing the low-temperature stress duration at days 1, 3, and 5. Similarly, the germination index values of maize plants in the V_B2_ and V_B12_ treatments were higher than CK. The average germination index of maize plants in V_B12_ treatment at days 1, 3, 5, and 7 were significantly higher by 59.05, 68.7, 40.41, and 49.19% than CK (Table 2).

**Table 2:** Germination rate and germination index of maize seeds germinated under low-temperature stress and vitamin treatment.

Treatment	Germination rate (%)				Germination index (%)			
	1 d	3 d	5 d	7 d	1 d	3 d	5 d	7 d
CK	41.13 ± 0.38c	41.25 ± 0.25c	36.50 ± 0.00b	31.00 ± 0.50b	41.13 ± 0.38c	41.25 ± 0.25c	36.50 ± 0.00b	31.00 ± 0.50c
V_B2_	60.84 ± 0.17ab	61.25 ± 0.35ab	50.84 ± 4.17a	50.84 ± 0.17a	60.84 ± 0.17ab	66.67 ± 0.34a	50.84 ± 0.17a	50.85 ± 0.15a
V_B12_	65.42 ± 0.09a	69.59 ± 0.92a	51.25 ± 0.25a	46.25 ± 0.25ab	65.42 ± 0.09a	69.59 ± 0.92a	51.25 ± 0.25a	46.25 ± 0.25ab
V_H_	55.00 ± 0.00ab	50.84 ± 0.17bc	40.42 ± 0.09ab	46.25 ± 0.75ab	55.00 ± 0.00ab	50.84 ± 0.17bc	40.42 ± 0.09ab	46.25 ± 0.75ab
V_PP_	51.25 ± 0.25bc	53.34 ± 0.34abc	50.00 ± 0.00ab	40.84 ± 0.17ab	51.25 ± 0.25bc	53.34 ± 0.34b	50.00 ± 0.00ab	40.84 ± 0.17b

Control (CK), vitamin B2 (V_B2_), vitamin B12 (V_B12_), biotin (V_H_), and nicotinic acid (V_PP_). Values with different letters in the same column are significantly different at *P*＜ 0.05 based on the *Duncan* test; data are represented as the mean of three replicates ± *S.E.*

### 3.3 Reactive oxygen species (H_2_O_2_ and O_2_^–^) and TBRS contents

**Fig. 1:**
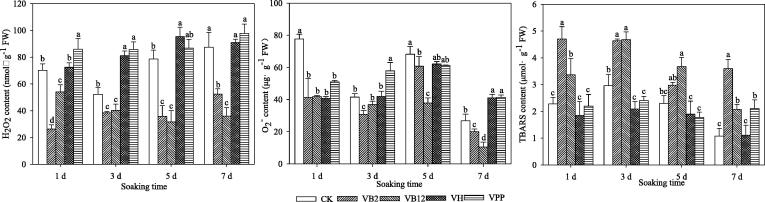
The contents of hydrogen peroxide (H_2_O_2_), superoxide anion (O_2_^–^) and thiobarbituric acid reactive substances (TBARS) in maize seedling leaves fresh weight (FW) after exogenous vitamin soaking of seeds at 5 °C. Different lowercase letters represent significant differences at *P* < 0.05; bars represent SE, Control (CK), vitamin B2 (V_B2_), vitamin B12 (V_B12_), biotin (V_H_), and nicotinic acid (V_PP_).

The H_2_O_2_ and O_2_^–^ contents in the V_B2_ and V_B12_ treatments were relatively low (Fig. 1). The H_2_O_2_ contents in the V_PP_ and V_H_ treatments were higher than other treatments of the group; however, after 5 days of soaking, the H_2_O_2_ contents in the V_B2_ and V_B12_ treatments were 54.36 and 59.69% lower than CK, respectively. Likewise, the O_2_^–^ contents in V_B2_ and V_B12_ treatments were lower, while significantly increased in V_H_ and V_PP_ by 49.46 and 50.48% compared to CK, respectively. The TBARS contents in both V_B2_ and V_B12_ treatments were 51.57 and 11.8%, 43.33 and 43.87%, 22.82 and 37.46%, and 70.01 and 48% higher than CK in low temperature at day 1, 3, 5, and 7, respectively. The results showed that the TBARS contents in V_B2_ were significantly higher than V_H_ and V_PP_ on days 1, 3, and 5 (*P* < 0.05).

### 3.4 Soluble osmolyte content

**Fig. 2:**
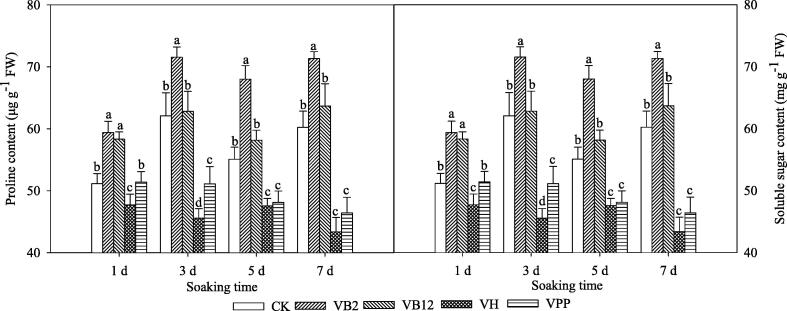
The proline and soluble sugar contents in maize seedling leaves fresh weight (FW) after exogenous vitamin soaking of seeds at 5 °C. Different lowercase letters represent significant differences at *P* < 0.05; bars represent SE, Control (CK), vitamin B2 (V_B2_), vitamin B12 (V_B12_), biotin (V_H_), and nicotinic acid (V_PP_).

The proline contents in the V_B2_ and V_B12_ treatments were higher than other treatments and the lowest with CK after days 1 and 3. The proline contents in V_B2_ (32.72, 42.27, 9.85 and 25.96%) and V_B12_ (22.53, 40.11, 8.49 and 3.15%) higher at day 1, 3, 5, and 7 than CK, respectively. The proline contents in the V_H_ and V_PP_ treatments were lower than that in CK on days 5 and 7. The soluble sugar contents in the V_B2_ and V_B12_ were higher than CK on days 1, 3, 5, and 7. These results showed that the increase in the sugar contents of the V_B2_ and V_B12_ treatments was higher than V_H_ and V_PP_ treatments (Fig. 2).

### 3.5 Antioxidant enzyme activities

**Fig. 3:**
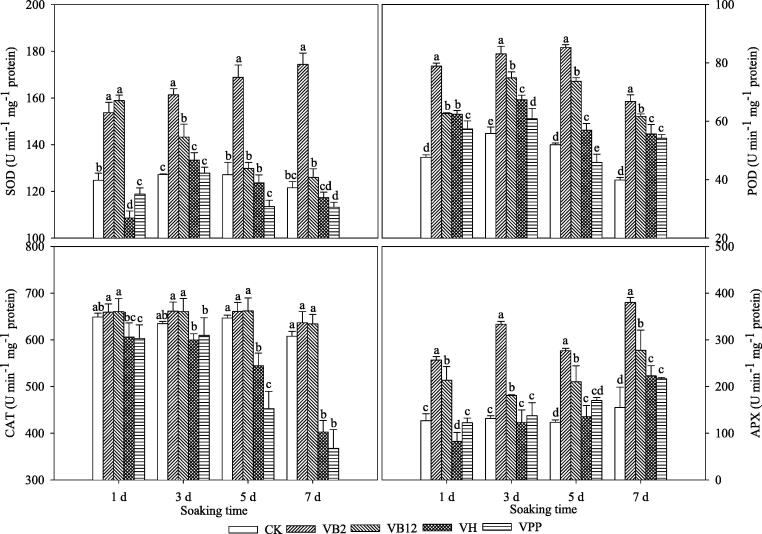
The superoxide dismutase (SOD), peroxidase (POD), catalase (CAT) and ascorbate peroxidase (APX) activities in maize seedling leaves fresh weight (FW) after seeds were soaked in exogenous vitamins at 5 °C. Different lowercase letters represent significant differences at *P* < 0.05; bars represent SE, Control (CK), vitamin B2 (V_B2_), vitamin B12 (V_B12_), biotin (V_H_), and nicotinic acid (V_PP_).

The activities of antioxidant enzymes in V_B2_ and V_B12_ treatments were higher than CK, V_H,_ and V_PP_ treatments (Fig. 3). The SOD activity in the V_B2_ is 23.22, 26.82, 32.79, and 43.44%, and in V_B12_ is 27.35, 12.53, 2.11, and 3.62% higher on days 1, 3, 5, and 7 compared to CK, respectively. Compared to CK, the V_H_ SOD activity was 12.93, 2.76, and 3.44%, and for V_PP_ was 4.79, 10.73, and 6.87% lower on day 1, 5, and 7, respectively.

The POD activity showed a similar change under different growing stages. The V_B2_ treatment had the highest POD activity compared to other treatments of the group, suggesting that the POD activity was 39.61, 32.78, 39.08, and 40.31% higher on day 1, 3, 5, and 7 than CK, respectively. The V_PP_ treatment exhibited slightly inhibited POD activity and was at day 5 found 13.08% lower than CK.

The CAT activities in the V_B2_ and V_B12_ treatments were relatively high, and the V_H_ and V_PP_ treatments had relatively low CAT activities. The results suggested that the CAT activities were 6.64, 5.52, 15.79, and 33.76% lower for V_H_ while 7.08, 3.98, 29.98, and 39.48 for lower for V_PP_ treatment at day 1, 3, 5, and 7 than CK, respectively.

The vitamin treatments had different effects on APX activity. Whereas the APX activity was also significantly increased with V_B2_ and V_B12_. The APX activity for V_B2_ was (102.63, 153.16, 124.32, and 145.16%) and for V_B12_ was (68.42, 37.34, 70.27, and 79.03%) higher at day 1, 3, 5, and 7 than CK, respectively. The V_H_ and V_PP_ treatments inhibited APX activities, and the effect was not significant; meanwhile, it resulted in 34.73 and 3.29% lower than CK on day 1.

### 3.6 Ascorbic acid content

**Fig. 4:**
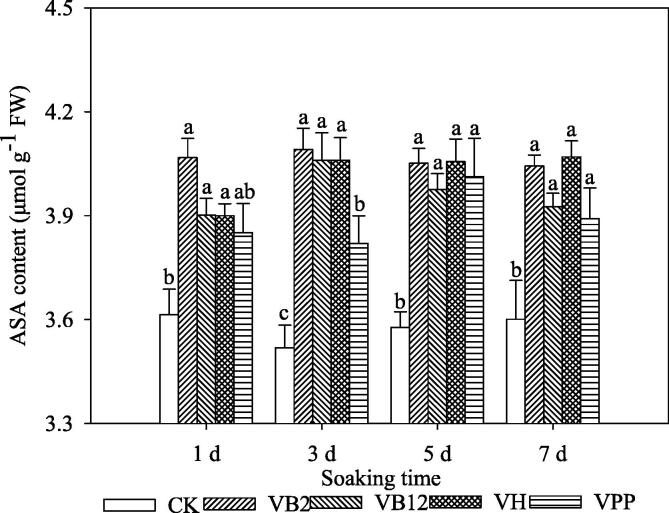
The ascorbic acid (ASA) content in maize seedling leaves fresh weight (FW) after seeds were soaked in exogenous vitamins at 5 °C. Different lowercase letters represent significant differences at *P* < 0.05; bars represent SE, Control (CK), vitamin B2 (V_B2_), vitamin B12 (V_B12_), biotin (V_H_), and nicotinic acid (V_PP_).

The results showed that the ASA contents in the vitamin treatments increased with time intervals (Fig. 4). The ASA contents in the V_B2_ and V_B12_ treatments were higher than CK; however, the ASA were non-significant among the four vitamin's treatment.

## 4 Discussion

Vitamins application to seeds regulates plant growth and antioxidant systems (Chinnusamy et al., 2007). Antioxidant capacity and various physiological processes of plants are negatively affected by low-temperatures which could be regulated using exogenous vitamins application (Savitch et al., 2009). The vitamin concentrations in soil are variable, and therefore a suitable treatment strategy should be used to protect plants from pathogens, particularly at low-temperatures.

The prolonged stress, particularly at low-temperature had increased the H_2_O_2_ content but decreased the O_2_^–^ contents in the plant's stress responses (Zhang et al., 2019). Disturbances in the metabolism of antioxidants with the capacity to quench/scavenge ROS led to alterations in stress responses. These factors can disrupt plant cell water balance and damage plant growth. Chilling stress affects water utilization by reducing water transportation and stomatal restriction, increasing ROS content, damaging membrane lipids, proteins, and nucleic acids, and hence causing cell death (Boubakri et al., 2013). The cell's substance infiltered to the surrounding environment due to damaged membrane and increased cell conductivity. This study indicated that chilling stress severely damaged the active oxygen metabolism of leaves in the seedling stage. The TBARS content in the V_B2_ and V_B12_ treatments was relatively high, which may be due to the enhancement of plants' oxidative stress. Thus, soaking seeds with exogenous vitamins can reduce the damage caused by cell membrane lipid peroxidation and improve plants' antioxidant capacity. Our results are supported by the finding of Aroca et al. (2003), who reported that vitamins enhance the antioxidant of crops and decrease membrane damage.

The production of different compatible solutes, commonly known as osmotic protectors, is also an important strategy for plant resistance to abiotic stress (Metwali et al., 2015, Pfannschmidt and Munné-Bosch, 2013). Previous researchers reported that osmotic protectors, including soluble protein, proline, and total free amino acids, protect plants from stress by contributing to cell osmotic regulation, enzyme and protein stabilization, ROS detoxification, and the integrity of protective membranes (Bartels and Sunkar, 2005, Liu et al., 2018). As a kind of osmotic protective agent, proline maintains cells expansion under stress conditions (Tiwari et al., 2010). It promotes the synthesis of essential proteins, which are necessary for the stress response (Iyer and Caplan, 1998). Our results showed that the leaf proline and soluble sugar contents changed under low-temperature stress. Substances accumulated and altered the osmotic equilibrium of cells in the V_B2_ and V_B12_ treatments; these results are in line with the finding of Wang et al. (2011). The leaf proline and soluble sugar contents in the V_H_ and V_PP_ treatments decreased gradually under low-temperature stress. This result suggested that the leaves might have accumulated more osmolytes to facilitate water transport.

Low-temperature stress can result in ROS accumulation in cells, damaging the dynamic equilibrium of ROS in plants (Suzuki et al., 2012), and increased the antioxidant defense system's activity to control the damage incurred by ROS accumulation. The previous study reported that exogenous vitamins impact the growth and development of plants (Heidarvand and Amiri, 2010), especially in terms of crop yield (Finch-Savage and Leubner-Metzger, 2006, Wang et al., 2018), morphological formation (Mollard and Insausti, 2009), and antioxidant enzyme activities (Freitas and Takaki, 2000). These results indicate that different types of vitamins have specific functions for eliminating ROS. Therefore, efficient antioxidant activity does not always reveal the upregulation of all antioxidant enzyme activities (Abogadallah, 2010, Ahmad et al., 2020a).

In the current study, the generation of ROS by low-temperature stress exceeded ROS removal capacity by the antioxidant defense system in the leaves, which may cause severe damage in the plant leaves (Sekmen et al., 2012, Xu et al., 2014). The increase in CAT activity in leaves is due to ROS accumulation. Our results are supported by previous studies, which indicated the affinity of CAT toward H_2_O_2_ is weak (Willekens et al., 1997); therefore, lower H_2_O_2_ concentrations are not physiologically acceptable levels.

A previous study showed that oxidative damage is the result of excessive accumulation of ROS under stress conditions (Naeem et al., 2018). It has been reported that H_2_O_2_ is stable in vivo compared to other ROS molecules in plants (Reth, 2002), and H_2_O_2_ is a signaling molecule with a tremendous impact on plant growth and development (Khan et al., 2018). The production of ROS is a sign of successful recognition of infection and activation of plant defenses. Hydrogen peroxide-induced lipid peroxidation of the cell membrane is usually measured as the content of TBARS. Plants have an effective antioxidant (enzymatic/nonenzymatic) defense mechanism, including SOD, POD, CAT, and APX activities (Cui et al., 2017, Wang et al., 2016). Super oxide dismutase is the key enzyme that regulates O_2_^–^ in leaves, whereas CAT and APX regulate H_2_O_2_ accumulation and reduce it to H_2_O. Initial treatment of plants containing H_2_O_2_ caused oxidative stress by disrupting ROS cell homeostasis and the ROS-dependent signaling network that enhances the accumulation of latent defense proteins (Ahmad et al., 2020a, Ahmad et al., 2019). However, H_2_O_2_ increased in V_H_ and V_PP_ treatments at days 3 and 5, which suggests reducing antioxidant enzymatic activity in tissues.

The leaf SOD, POD, and APX activities were increased by soaking with V_B2_ and V_B12_. This demonstrated that catalase activity decreased with decreasing H_2_O_2_ content in leaves, while antioxidant enzymatic activities increased with V_B2_ and V_B12_ treatments under low-temperature stress. Reactive oxygen species scavenging enzymes and modulation of physiological processes due to higher stress responses (Borges et al., 2014). Moreover, seed soaking with exogenous vitamins increased the ASA content and reduced ROS generation, thus alleviating damage to the cell membrane system. These results are consistent with previous studies that showed that vitamin treatment could reduce cell damage and enhance cell membrane stability (Tang et al., 2005, Troesch et al., 2012).

Seedling growth is usually viewed as yielding information for living plants. In our study, plants' growth parameters were significantly affected by vitamins, especially V_B2_ and V_B12_. The germ and radicle lengths and fresh weights of seedlings in the V_H_ and V_PP_ treatments were lower than V_B2_ and V_B12_ treatments. The V_B2_ and V_B12_ treatments improved the germination rate, index, and root-shoot ratio; however, V_H_ and V_PP_ decreased the root-shoot ratio at day 3. Our results showed that V_B2_ and V_B12_ were beneficial for seedling growth under low-temperature stress than other treatments and CK.

Exogenous vitamins increase plant resistance to abiotic stresses, such as chilling stress (Guler et al., 2016, Zhang and Gan, 2016), drought, and saline conditions, by improving antioxidant defense capabilities in plants (Ahmad et al., 2015, Hashem et al., 2014). Different plant cultivars have different mechanisms for the inhibition and promotion of growth. In this study, seed soaking with exogenous vitamins improved SOD, POD, CAT, and APX enzymatic activities. Similarly, the concentrations of soluble sugars, proline, TBARS, and ASA increased while decreased H_2_O_2_, O_2_^–^, and ROS. These enzymatic activities increase antioxidant resistance, improve the water-absorbing capacity of cells, and enhance the resistance of seedlings' chilling stress. Exogenous vitamin applications in plant production are a valuable tool for protecting plants against low-temperature stress.

## 5 Conclusions

Maize seeds soaked in 100 mg of V_B2_ and V_B12_ per liter of distilled water solutions stimulated the leaves' physiological processes. These vitamins increased antioxidant enzymatic activities and alleviated peroxidation damage of membrane lipids, promoting seedling growth under low-temperature stress. The germ and radicle fresh weights of maize plants treated with V_H_ and V_PP_ slightly decreased compared with CK. Thus, the V_B2_ and V_B12_ can be an alternative to conventional treatment methods and should be adopted in future research on active oxygen metabolism and seedling growth under low-temperature stress.
